# Human paraoxonase 1 overexpression in mice stimulates HDL cholesterol efflux and reverse cholesterol transport

**DOI:** 10.1371/journal.pone.0173385

**Published:** 2017-03-09

**Authors:** Souade Ikhlef, Hicham Berrougui, Olivier Kamtchueng Simo, Echarki Zerif, Abdelouahed Khalil

**Affiliations:** 1 Research Centre on Aging, Sherbrooke, Quebec, Canada; 2 Department of Biology, Polydisciplinary Faculty, University Sultan Moulay Slimane, Beni Mellal, Morocco; 3 Department of Pediatric, Immunology Division, Centre de Recherche Clinique CHUS, University of Sherbrooke, Sherbrooke, Quebec, Canada; 4 Department of Medicine, Geriatrics Service, Faculty of Medicine and Health Sciences, University of Sherbrooke, Sherbrooke, Quebec, Canada; Beijing Key Laboratory of Diabetes Prevention and Research, CHINA

## Abstract

This study was aimed to investigate the effect of human PON1 overexpression in mice on cholesterol efflux and reverse cholesterol transport. PON1 overexpression in PON1-Tg mice induced a significant 3-fold (p<0.0001) increase in plasma paraoxonase activity and a significant ~30% (p<0.0001) increase in the capacity of HDL to mediate cholesterol efflux from J774 macrophages compared to wild-type mice. It also caused a significant 4-fold increase (p<0.0001) in the capacity of macrophages to transfer cholesterol to apoA-1, a significant 2-fold (p<0.0003) increase in ABCA1 mRNA and protein expression, and a significant increase in the expression of PPARγ (p<0.0003 and p<0.04, respectively) and LXRα (p<0.0001 and p<0.01, respectively) mRNA and protein compared to macrophages from wild-type mice. Moreover, transfection of J774 macrophages with human PON1 also increased ABCA1, PPARγ and LXRα protein expression and stimulates macrophages cholesterol efflux to apo A1. In vivo measurements showed that the overexpression of PON1 significantly increases the fecal elimination of macrophage-derived cholesterol in PON1-Tg mice. Overall, our results suggested that the overexpression of PON1 in mice may contribute to the regulation of the cholesterol homeostasis by improving the capacity of HDL to mediate cholesterol efflux and by stimulating reverse cholesterol transport.

## Introduction

Atherosclerosis is the primary factor involved in cardiovascular disease events. It is initiated by the formation of foam cells following the engorgement of macrophages with oxidized LDL and their deposition in arteries, leading to arterial stenosis. Epidemiological studies have shown that there is an inverse relationship between HDL concentration and the risk of atherosclerotic lesion formation and cardiovascular disease events. This aroused interest to develop pharmacological agents that increase HDL concentrations. While such agents do significantly increase HDL concentrations, their effect on cardiovascular protection has been very disappointing [1,2]. On the other hand, there is increasing evidence to suggest that HDL function may be more important than HDL concentration in protecting against cardiovascular diseases [3]. The idea that function plays an important role in the anti-atherosclerotic effect of HDL has increased interest in exploring factors that may improve HDL functionality.

The functionality of HDL relates to their capacity to exert an anti-atherosclerotic effect, *i*.*e*., anti-inflammatory and antioxidant activities, and the capacity to mediate cellular cholesterol homeostasis via the reverse cholesterol transport (RCT) process. RCT, which ensures the uptake of cholesterol from peripheral cells and its transport back to the liver for excretion in the bile and ultimately in the feces, is believed to be the main anti-atherogenic function of HDL [4]. Cholesterol efflux from macrophages constitutes the first and rate-limiting step of RCT [5]. Interestingly, cholesterol efflux from HDL is significantly and inversely correlated with carotid intima-media thickness and the incidence of cardiovascular events, even after adjusting for the concentration of HDL [3,6].

Apolipoprotein A-1 (ApoA-1) is the major apoprotein forming HDL and plays an important role in cholesterol metabolism. ApoA-1 initiates cholesterol efflux from macrophages by reacting specifically with ABCA1 to generate nascent HDL particles, which are then transformed into mature spherical HDL particles during their enrichment with cholesterol [7]. Several other proteins associated to HDL, including paraoxonase 1 (PON1), may also have a significant effect in the regulation of the anti-atherosclerotic activities of HDL and particularly their capacity to mediate cholesterol homeostasis [8].

PON1 is a 355-amino-acid, 43-kDa polypeptide that is synthesized by the liver and then released into the circulation where it is exclusively associated with HDL [9]. While the physiologic role of PON1 is not clear, it has been suggested that it hydrolyzes oxidized phospholipids in oxLDL, abolishing their biological activities [10]. Loued et al. recently showed that PON1 possesses phospholipase-A2-like activity that allows it to hydrolyze oxidized phospholipids at the sn-*2* position, contributing to the formation of lysophosphatidylcholine [10].

While increasing evidence suggests that PON1 possesses atheroprotective activity, the mechanism involved has not been clearly identified. Recent meta-analyses highlighted the association between lower plasma PON1 activity and an increased risk for coronary artery disease (CAD) [11,12]. Animal studies have also confirmed the anti-atherosclerotic effect of PON1 [13,14]. These studies have shown that PON1 knockout mice are more susceptible to atherosclerosis and that overexpression of PON1 increases the resistance of the mice to atherosclerosis [13,14]. The atheroprotective effect of PON1 may be related to its antioxidant activity and its capacity to modulate oxidative stress [15]. Interestingly, several studies, among ours, have shown that PON1 regulates the cholesterol efflux from macrophage to HDL [8,16–18]. Berrougui et al. [8] demonstrated that this effect of PON1 is mediated by increasing the associative binding of apoA-1 and HDL to macrophages [17,18]. It has also been suggested that PON1 stimulates ABCA1 expression on macrophages, increasing cholesterol efflux to HDL. Given that cholesterol efflux from macrophages to HDL is an important step in cholesterol homeostasis, its regulation by PON1 may be a pathway by which PON1 protects against CAD. The aim of the present study was to examine the effect of PON1 overexpression on cholesterol homeostasis in vivo by measuring RCT and to determine the mechanism through which PON1 mediates this effect.

## Materials and methods

### Chemicals

Sodium dodecyl sulfate (SDS), and Tween 20 were from Fisher Scientific (Canada). Thioglycolate broth, poly(ethylene glycol) (PEG), ethylenediaminetetraacetic acid disodium salt dehydrate (EDTA), HCl, and 8-(4-chlorophenylthio)adenisine3’:5’-cyclic monophosphate (cAMP) were from Sigma-Aldrich (USA). Acrylamide bis-acrylamide was from Amresco (USA). TEMED and ammonium persulfate were from Bio-Rad (Canada). RIPA buffer was from Cell Signaling Technology (USA). The pCMV3-PON-OFPSpark plasmid was obtained from Sino Biological Inc (Beijing. China) and HDLc measurement kit from abcam (abcam, ON, Canada). Primers were from Integrated DNA Technologies, Inc. (USA). Cholesterol, (1, 2- ^3^H (N)) (^3^H-cholesterol), and Western Lightning^®^ Plus-ECL were from Perkin Elmer (Canada).

### Animals

Eight-week-old male wild-type (WT) C57BL/6 mice were purchased from Charles River Laboratories (QC, Canada). The transgenic PON1 (PON1-Tg) mice were a generous gift from Dr. Yin Tintut (University of California, LA, USA). This study was carried out in strict accordance with the recommendations in the Guide for the Care and Use of Laboratory Animals of the Canadian Council on Animal Care in science. The protocol was approved by the Committee on the Ethics of Animal Experiments of the University of Sherbrooke (Permit Number: 263-11R). All efforts were made to minimize suffering.

### Cell cultures

J774 macrophage-like cells were (American Type Culture Collection, Manassas, VA) maintained in Dulbecco's Modified Eagle's Medium (DMEM) supplemented with 10% heat-inactivated fetal bovine serum (FBS), 100 U/mL of penicillin and 100 U/mL of streptomycin in humidified atmosphere (5% CO_2_) at 37°C. Primary peritoneal macrophages were obtained using the thioglycholate method as previously described by Linton et al. [19]. Briefly, 4 mL of 4% (w/v) thioglycolate medium were injected intraperitoneally and five days later macrophages were harvested by injecting 10 mL of cold DMEM into the abdominal cavity [19]. The cells were pelleted, re-suspended in DMEM supplemented with 5% FBS, and plated at a density of 10^5^ cells/cm^2^. Cells were allowed to adhere for 4 h, and non-adherent cells were removed by rinsing with DMEM. The adherent cells (macrophages) were used for the experiments described below.

### Measurement of in vitro cholesterol efflux

J774 macrophages were used at basal condition or after enrichment with ABCA1 transporter. For this later, J774 macrophages were incubated with cAMP (0.3 mM), in serum-free medium containing 1% BSA, for 16 h to produce ABCA1-enriched cells. The cells were collected by centrifugation (350 x g for 10 min) and were lysed in 0.1 M NaOH.

HDL were obtained from mouse plasma following the precipitation of apolipoprotein B-containing lipoproteins in the presence of 20% polyethylene glycol (PEG). Cholesterol efflux was performed as previously described [20]. Briefly, J774 macrophages, J774 macrophages transfected with hPON1 and peritoneal macrophages obtained from wild type and PON1-Tg mice were radiolabeled for 24 h with 2 μCi/mL of [^3^H]-cholesterol in DMEM. Cholesterol efflux was assessed by incubating the radiolabeled J774-macrophages for 4 h at 37°C with 50 μL/mL of apolipoprotein B-depleted plasma (PEG-treated plasma from wild-type or PON1-Tg mice)]; J774 macrophages transfected with hPON1 or MPM with 50 μg/mL of ApoA-1. The supernatants and the adherent cells (macrophages) were separated and prepared for [^3^H]-cholesterol quantification as previously described by Loued et al. [21]. A liquid scintillation counter was used to determine [^3^H]-cholesterol level (counts/min or cpm) in both the supernatant and in macrophages (lysates). Cholesterol efflux was calculated using the following formula: (radioactivity (cpm) in supernatant/radioactivity (cpm) in cells + medium) x 100.

### HDLc analysis

HDLc plasma concentration was determined by enzymatic colorimetric assay according to the manufacturer’s protocol using a commercially available test kit (ab65390 from abcam, ON, Canada). The absorbance was read at 570 nm using Hitachi UH 5300 spectrophotometer.

### Measurement of PON1 paraoxonase activity

PON1 paraoxonase activity was determined by the measurement of the hydrolysis rate of paraoxon (O, O-diethyl-O-p-nitrophenylphosphate; Sigma) as previously described [22]. Paraoxon was used at concentration 5.5 mM in 100 mM Tris-HCl buffer (pH 8.0) containing 2 mM CaCl_2_ and the reaction was initiated by the addition of 10 μl of plasma. The rate of 4-nitrophenol formed was measured at 412 nm. One unit of paraoxonase activity was defined as 1 nmole 4-nitrophenol formed per minute (molar extinction coefficient of 13 893.75 M^-1^cm^-1^).

### J774 macrophages transfections

J774 macrophages (10^6^) were transfected with human PON1. Briefly, the J774 cells were seeded in six-well culture plates and cultured until the cells reached 70–80% confluence. The pCMV3-PON1-OFPSpark plasmid was transfected into the J774 cell lines by Lipofectamine 2000 (Invitrogen, Carlsbad, CA, USA) according to the manufacturer’s protocol. J774 macrophages transfected with cDNA insert lacking the human PON1 gene were used as control. The pCMV3-PON1-OFPSpark plasmid was purchased from Sino Biological Inc. (Beijing, China; HG13083-ACR). The transfection efficiency was around 80%.

### Reverse cholesterol transport measurement

J774 macrophages grown in DMEM supplemented with 10% FBS were radiolabeled with 2 μCi/mL of [^3^H]-cholesterol [20]. After 12 h equilibration in DMEM supplemented with 1% FBS and 1% BSA, the radiolabeled macrophages were washed with PBS and 8 x 10^6^ cells containing 3.8 × 10^6^ counts per minute [cpm] in 0.5 mL DMEM and were injected into the peritoneal cavities of the wild-type and PON1-Tg mice.

Blood was collected after 6, 24, and 48 h, and the plasma was isolated for the measurement of [^3^H]-cholesterol level. Feces were collected during the 48-h period and the cholesterol and bile acids were extracted. The mice were euthanized after 48 h and their livers were used for radioactivity measurement (total liver [^3^H]-cholesterol) [23]. The results are expressed as the percent of the radioactivity injected recovered in plasma, liver, and feces (in cholesterol and bile acids fractions) and the plasma volume was estimated as 3.5% of the animal weight.

### Western blot analysis

Peritoneal macrophages from wild-type and PON1-Tg mice were lysed in RIPA buffer. Protein were separated on 10% SDS-PAGE gels and immunobloted with ABCA1, PPARγ, LXRα or β-actin primary antibodies (Abcam, USA) as previously described by Ikhlef et al. [24].

### Analysis of mRNA by real time quantitative PCR

Total RNA from mouse peritoneal macrophages (MPM) from wild-type and PON1-Tg mice was prepared using EZ-10 Spin Column Genomic RNA Miniprep kits (Bio Basic Inc., Canada) according to the instruction given by the manufacturer. Samples of 2 μg of total RNA were used for RT-PCR [8]. The typical PCR reaction consisted of 40 cycles (95°C for 40 s, 56°C for 40 s, and 72°C for 40 s). The resulting products were resolved by 2% agarose gel electrophoresis and detected by ethidium bromide staining. *ABCA1*, *PPARγ*, and *LXRα* gene expression was normalized to the corresponding amount of β-actin. The relative quantities of target genes were determined using the ΔΔC_t_ method as previousely described by Ikhlef et al. [24]. The primer sets are listed in Table 1.

**Table 1:** Primer sequences for the real-time quantitative PCR.

Genes	Forward primers (50 to 30)	Reverse primers (50 to 30)
**ABCA1**	5’-AGCCAGAAGGGAGTGTCAGA-3’	5’-CATGCCATCTCGGTAAACCT-3’
**PPARγ**	5’-GGTTGACACAGAGATGCCATTCTGGCC-3’	5’-AAGCTTCCATCGGATGGTTCTTCG-3’
**LXRα**	5’-AGGAGTGTCGACTTCGCAAA3’	5’-CTCTTCTTGCCGCTTCAGTTT-3’
**β-actin**	5’-GTGGGCCGCTCTAGGCACCAA-3’	5’CTCTTTGATGTCACGCACGATTTC-3’

ABCA1, ATP-binding cassette A1; PPARγ, peroxisome proliferator-activated receptor gamma; LXRα, liver X receptor alpha

### Statistical analysis

Values are expressed as means ± SEM. Mean values were compared using the Student’s *t* test. P values less than or equal to 0.05 were considered significant. Statistical analyses were performed using GraphPad Prism software, version 6.0 (GraphPad Software, Inc., USA).

## Results

### Human Paraoxonase 1 (PON1) overexpression enhances plasma paraoxonase activity and J774 macrophage cholesterol efflux

**Fig 1:**
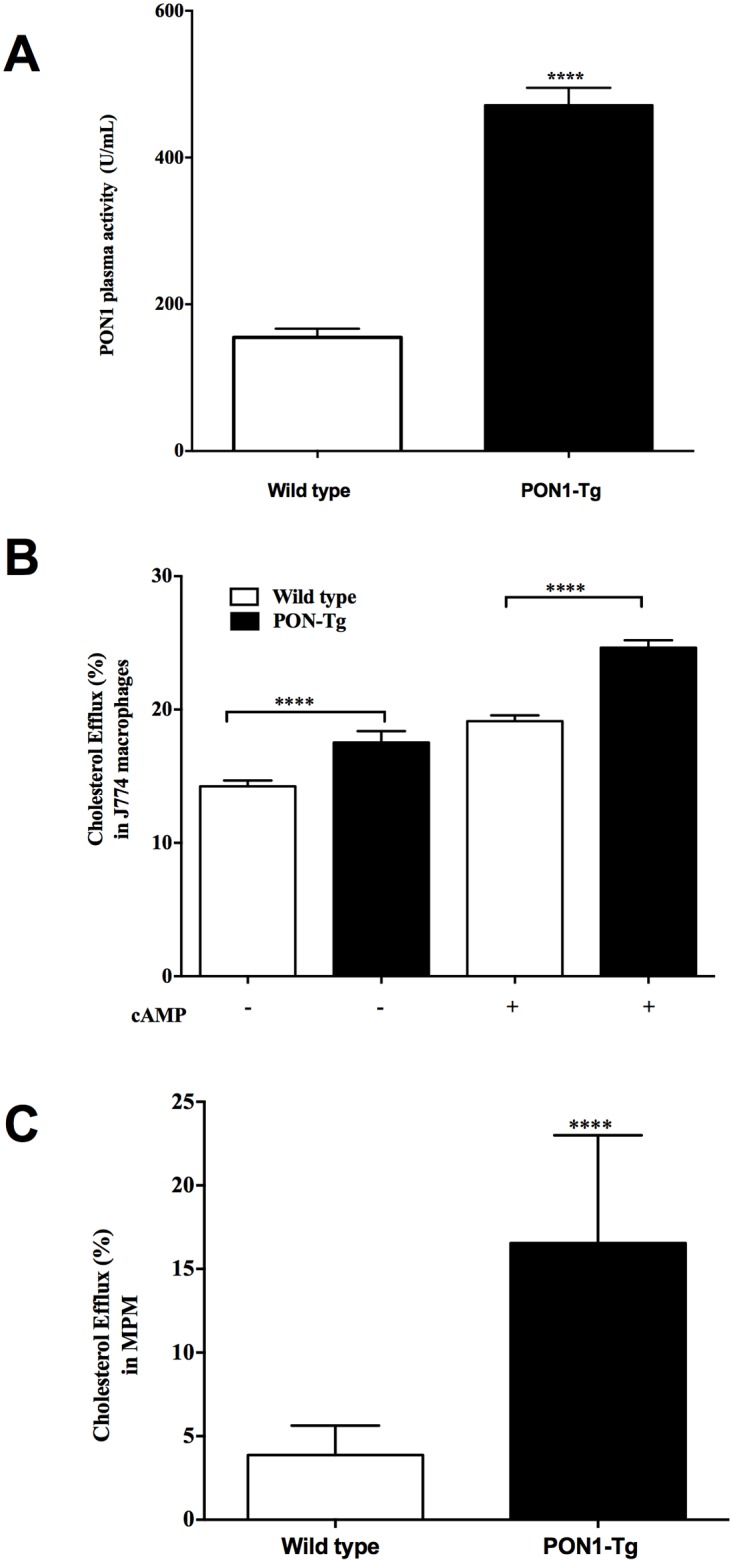
PON1 overexpression in mice enhances macrophage-derived cholesterol efflux. **(A)** Plasma was obtained from WT and PON1-Tg mice. Paraoxonase activity was determined by recording the increase in absorbance at 412 nm using paraoxon as the substrate. One unit of paraoxonase activity was defined as 1 nmole 4-nitrophenol formed per minute. **(B)** J774 macrophages (1 x 10^6^ cells/mL) were loaded with [^3^H]-cholesterol (2 μCi/mL) for 24 h and were incubated with or without cAMP (0.3 mM) for 12 h. The cells were subsequently incubated with 50 μL/mL of plasma from WT or PON1-Tg mice, which was then precipitated with PEG. **(C)** MPM from WT and PON1-Tg mice (1 x 10^6^ cells/mL) were loaded with [^3^H]-cholesterol (2 μCi/mL) for 24 h. Following a 12-h pre-incubation phase, the cells were incubated with 50 μg/mL of Apo-A1 for 4 h. Data are expressed as means ± SEM. n = 14 mice/group. ****p<0.0001.

Heparin-treated plasma was obtained from WT and PON1-Tg mice for the measurement of PON1 paraoxonase activity and HDL cholesterol (HDLc) concentration. As shown in Fig 1A, PON1-Tg mice exhibited significantly higher plasma paraoxonase activity (204% higher, p<0.0001) than WT mice. However, overexpression of PON1 in mice increases does not affect significantly the plasma HDLc; 113.0 ± 5.2 mg/dL for WT compared to 98.33 ± 4.7 mg/dL for PON1-Tg mice (p<0.102).

Previous studies have shown that in vitro enrichment of HDL with human PON1 improves the functionality of HDL by, in part, increasing their anti-inflammatory activity as well as their capacity to mediate macrophage-derived cholesterol efflux [8,10]. In the present study, we investigated the effect of the overexpression of human PON1 in mice on the capacity of HDL to mediate cholesterol homeostasis in vitro and in vivo and to elucidate the mechanism of this regulation. J774 macrophages were loaded with cholesterol and were labeled with [^3^H]-cholesterol (2 μCi/mL). ABCA1 overexpression by J774 macrophages was induced by incubation with a cAMP analog. Our results showed that HDL from PON1-Tg mice have a significantly higher capacity to mediate cholesterol efflux from J774 [^3^H]-radiolabeled macrophages than HDL from WT mice **(**Fig 1B**)**. The increase was significantly higher whether HDL were incubated with J774 macrophages under basal conditions (23% higher, p<0.0001) or with ABCA1-enriched J774 macrophages (29% higher, p<0.0001) (Fig 1B)

### Human PON1 overexpression stimulates cholesterol efflux from mouse peritoneal macrophages

Cholesterol efflux is a complex process that depends on the capacity of HDL to accept cholesterol from macrophages and on the liberation of excess cholesterol by macrophages. To determine whether PON1 overexpression regulates the capacity of macrophages to export excess cholesterol, we measured cholesterol efflux from MPM to apoA-1. MPM were isolated from PON1-Tg and WT mice. These MPM were radiolabeled with [^3^H]-cholesterol (2 μCi/mL) and incubated with apoA-1 (50 μg/mL) to initiate the efflux of cholesterol. As shown in Fig 1C, cholesterol efflux from MPM from PON1-Tg mice to apoA-1 was significantly higher than cholesterol efflux from MPM from WT mice (328% higher, p <0.0001).

### Human PON1 overexpression stimulates cholesterol efflux by up-regulating ABCA1 expression

**Fig 2:**
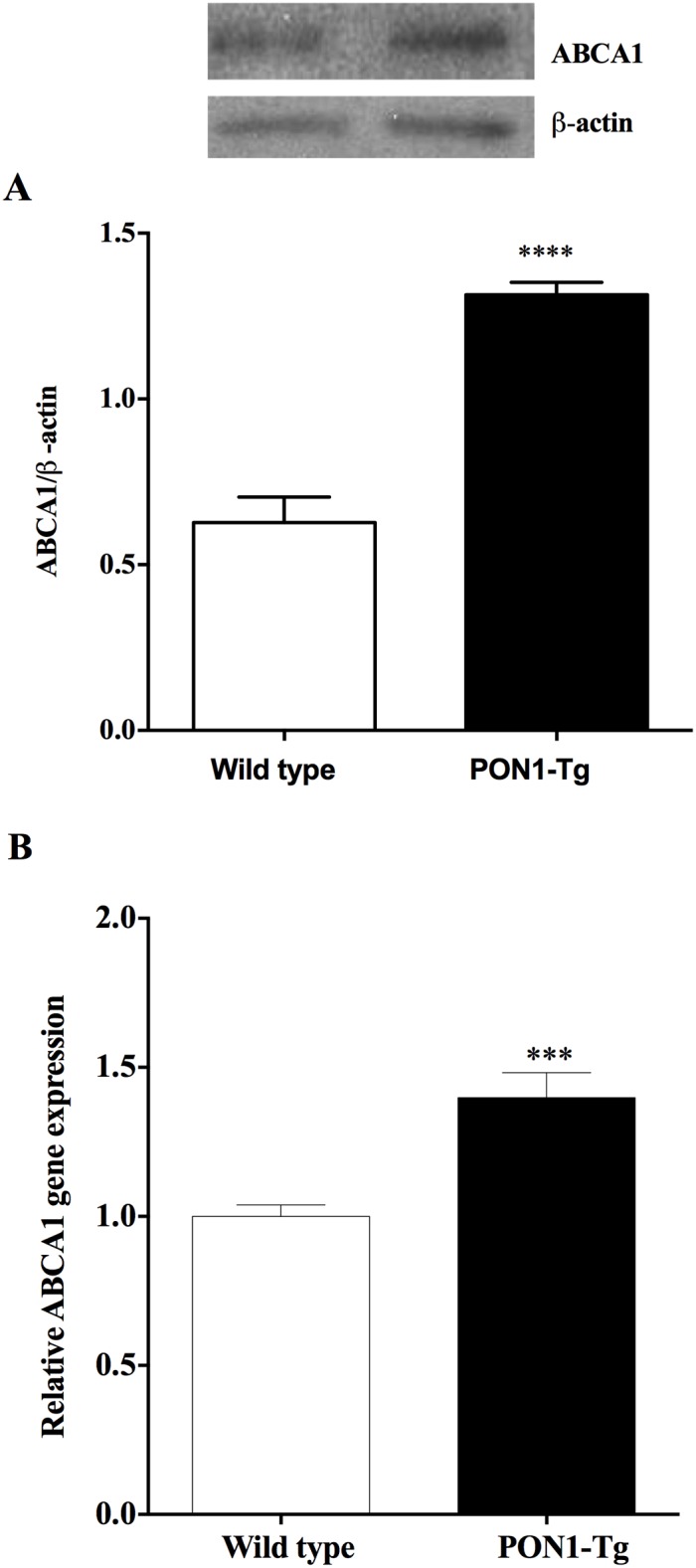
PON1 overexpression in mice up-regulates ABCA1 protein and RNA expression by MPM. ABCA1 protein and RNA expression by MPM from WT and PON1-Tg mice were determined by **(A)** Western blotting (n = 5 mice/group) and **(B)** RT-PCR (n = 4 mice/group), respectively. Data are expressed as means ± SEM. ***p = 0.0009, ****p<0.0001.

The ABCA1 transporter plays a significant role in the regulation of the efflux of cholesterol from macrophages to apoA-1. Previous studies by our laboratory have shown that the overexpression of ABCA1 transporter on J774 macrophages increases the capacity of these macrophages to export excess cholesterol to apoA-1 and HDL [24]. Given this, we determined whether the overexpression of PON1 in mice had an effect on ABCA1 levels in MPM. As shown in Fig 2A, MPM from PON1-Tg mice expressed significantly higher levels of ABCA1 transporter protein (109% higher, p<0.0001) and mRNA (82% higher, p = 0.0009) than MPM from WT mice **(**Fig 2B**)**.

### Human PON1 overexpression stimulates ABCA1 expression through the PPARγ/LXRα signaling pathway

**Fig 3:**
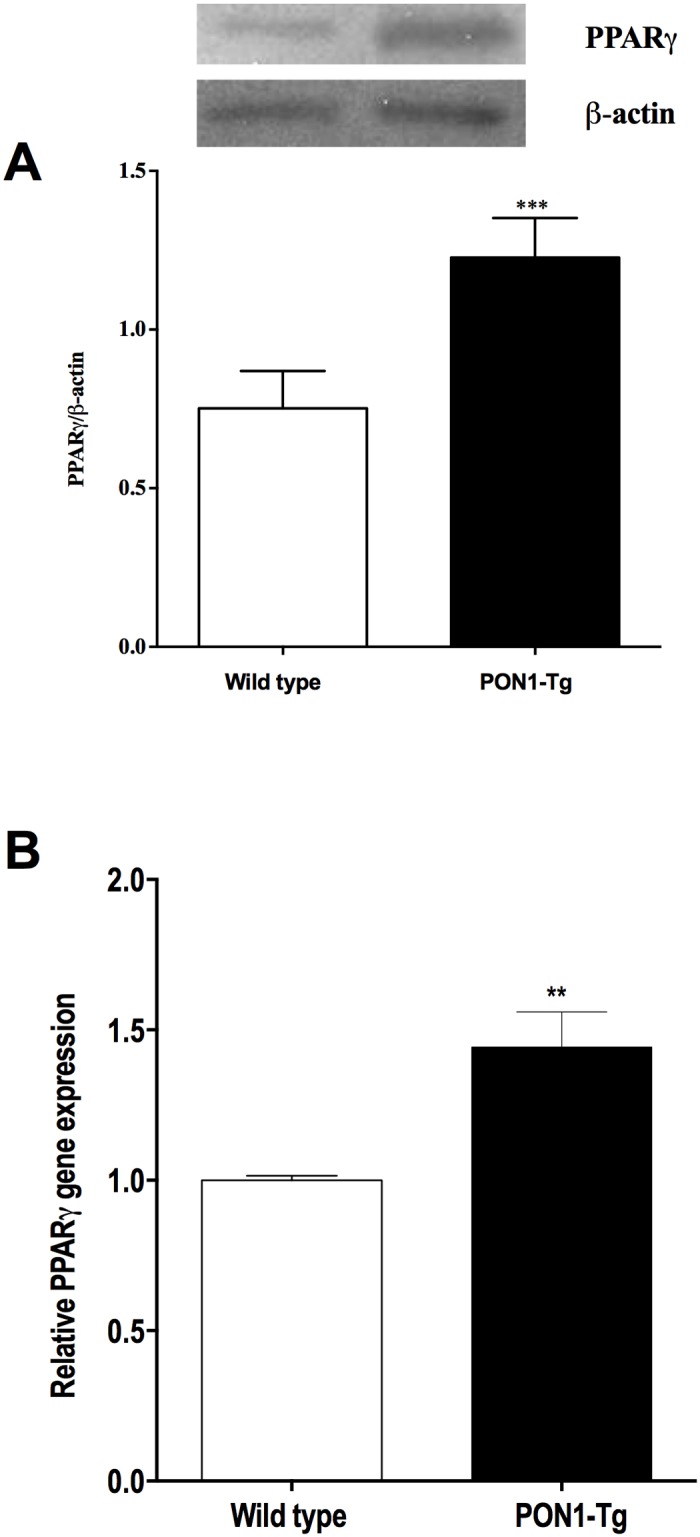
PON1 overexpression in mice up-regulates PPARγ protein and RNA expression by MPM. PPARγ protein and RNA expression by MPM from WT and PON1-Tg mice were analyzed by **(A)** Western blotting (n = 5 mice/group) and **(B)** RT-PCR (n = 4 mice/group), respectively. Data are expressed as means ± SEM. *p = 0.0451, ***p = 0.0003.

**Fig 4:**
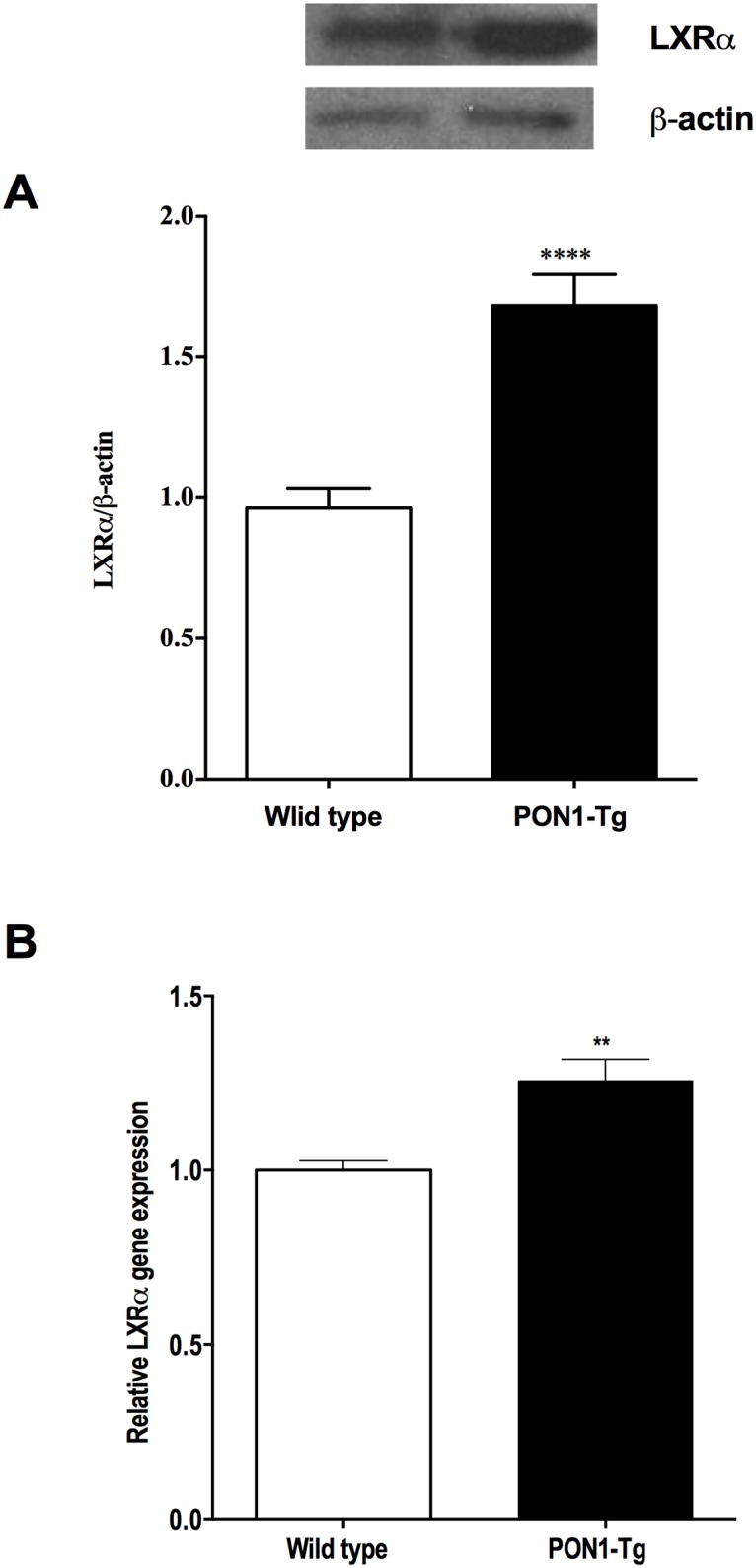
PON1 overexpression up-regulates LXRα protein and RNA expression. LXRα protein and RNA expression by MPM from WT and PON1-Tg mice were determined by **(A)** Western blotting (n = 5 mice/group) and **(B)** RT-PCR (n = 4 mice/group), respectively. Data are expressed as means ± SEM. *p = 0.0127, ****p<0.0001.

The expression of ABCA1 is transcriptionally regulated by nuclear transcription factor liver X receptor (LXR) and peroxisome proliferator-activated receptor (PPARγ). To gain further insight into the mechanism by which the overexpression of PON1 may stimulate ABCA1 expression, we measured the expression of PPARγ and LXRγ protein and mRNA in MPM from PON1-Tg mice and WT mice. Our results showed that MPM from PON1-Tg mice express significantly higher levels of PPARγ and LXRα protein (Figs 3A and 4A) and mRNA (Figs 3B and 4B) than MPM from WT mice.

### Human PON1 transfection into J774 macrophages increases cholesterol efflux

**Fig 5:**
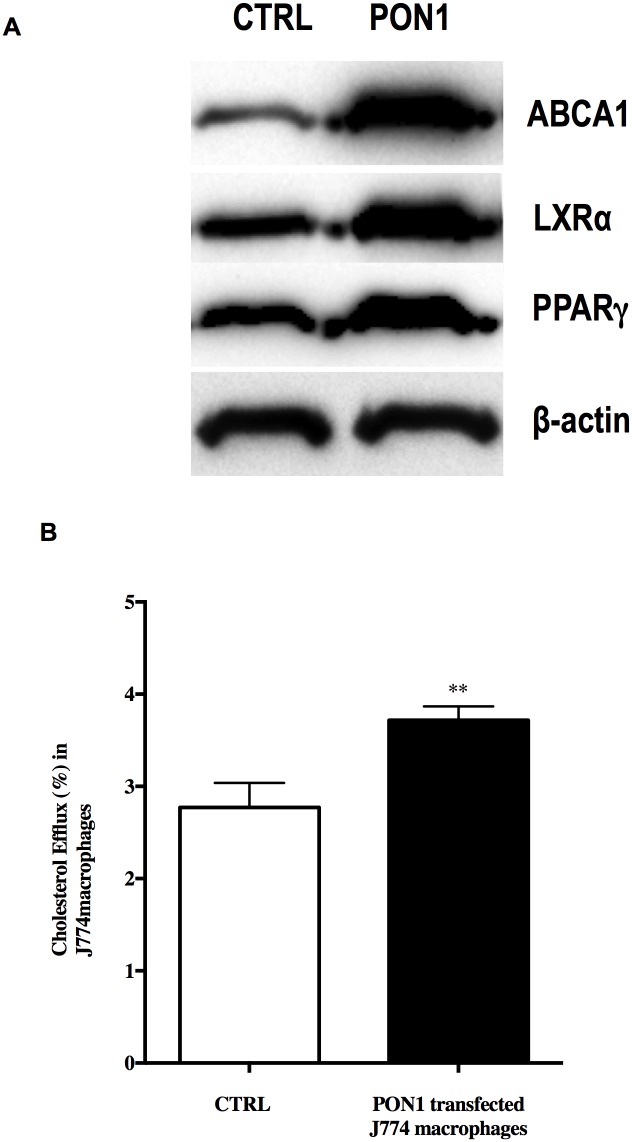
Transfection of J774 macrophages with human PON1 up-regulates ABCA1, LXRα and PPARγ protein expression and stimulates cholesterol efflux. **(A)** ABCA1, LXRα and PPARγ protein expression by J774 macrophages was determined by Western blot. (**B)** PON1 transfected J774 macrophages and control macrophages (CTRL) were loaded with [^3^H]-cholesterol (2 μCi/mL) for 24 h followed by incubation with 50 μg/mL of apoA-1 for 4 h. Data are expressed as means ± SEM, n = 3. **p<0.005.

To confirm the results obtained with MPM isolated from mice overexpressing PON1 we used J774 macrophages that were transfected with human PON1. Transfection of J774 macrophages with human PON1 induced a significant increase of the ABCA1 protein expression when compared to control J774 macrophages (Fig 5A). Moreover PPARγ and LXRα expression was also significantly increased in J774 transfected macrophages when compared to control macrophages (Fig 5A). This increase was accompanied by a significant enhancement of cholesterol efflux from PON1-transfected macrophages to apoA1 (Fig 5B) (p <0.005).

### Human PON1 overexpression increases Reverse Cholesterol Transport (RCT)

**Fig 6:**
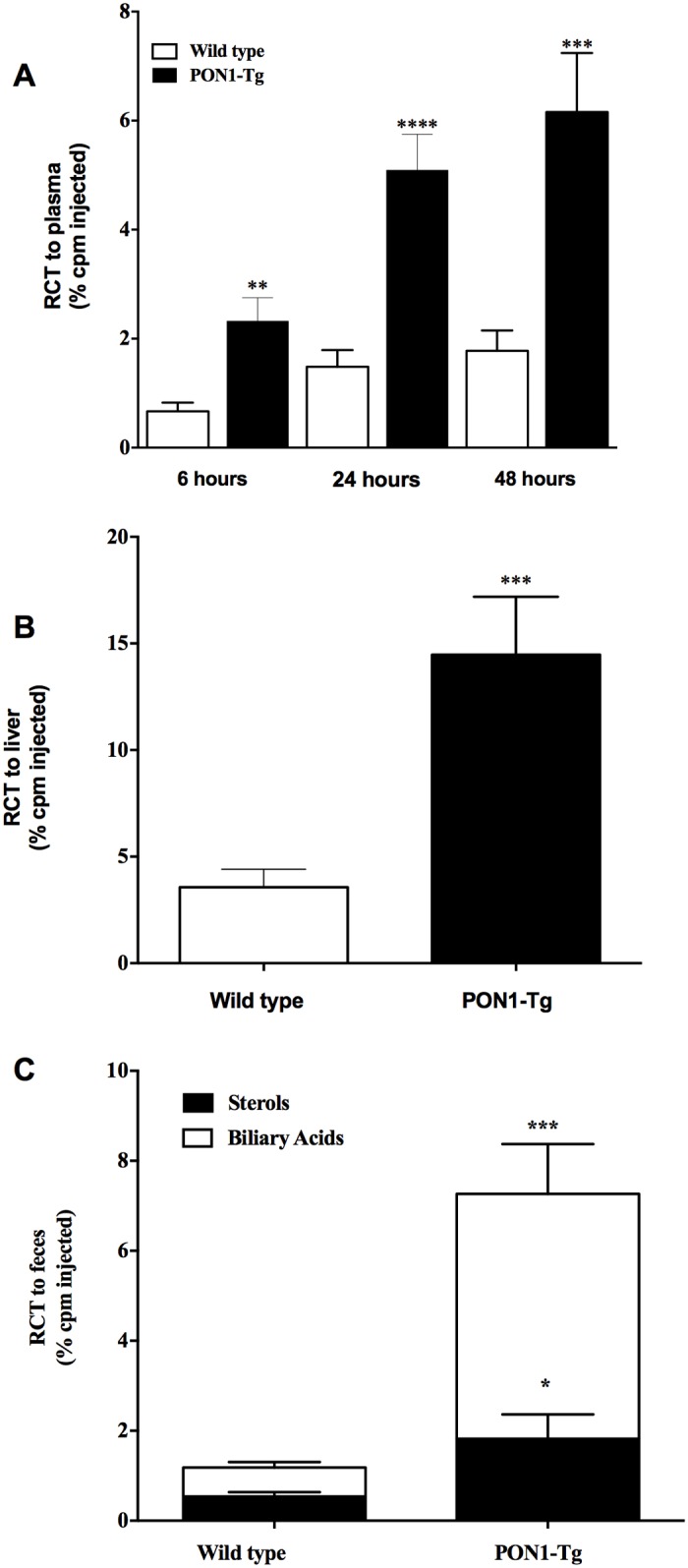
PON1 overexpression increases reverse cholesterol transport. [^3^H]-cholesterol-loaded J774 macrophages were injected intraperitoneally into WT and PON1-Tg mice. [^3^H]-cholesterol plasma levels were measured at 6, 24, and 48 h post-injection. Feces were collected continuously from 0 to 48 h post-injection. **(A)** Time course of [^3^H]-cholesterol distribution in the plasma, **(B) [**^3^H]-cholesterol recovery in the liver after 48 h, and (**C) [**^3^H]-cholesterol recovery in the feces as sterols and biliary acid. Data are expressed as means ± SEM. n = 14 mice/group. **p = 0.0013, ***0.0007<p<0.0008, ****p<0.0001.

We also explored the effect of human PON1 overexpression in mice on cholesterol homeostasis by measuring RCT. [^3^H]-cholesterol-loaded J774 macrophages were injected into the peritoneal cavities of PON1-Tg and WT mice. [^3^H]-cholesterol counts in the plasma were expressed as a percentage of the total [^3^H]-cholesterol injected (Fig 6A). [^3^H]-cholesterol counts in the plasma of PON1-Tg mice were significantly higher at all time points (6, 24, and 48 h) than those of WT mice (more than 240% higher (p = 0.0013) at 6 h, 343% higher at 24 h (p<0.0001), and 347% higher at 48 h (p = 0.0007). [^3^H]-cholesterol counts in the livers of PON1-Tg mice were 305% (p = 0.0007) higher after 48 h than in the livers of WT mice (Fig 6B). The measurement of [^3^H]-cholesterol counts in the feces showed that human PON1 overexpression significantly increases (514% higher, p = 0.0008) the fecal excretion of macrophage-derived cholesterol by PON1-Tg mice compared to WT mice (Fig 6C).

## Discussion

Atherosclerosis is the primary risk factor for stroke, myocardial infarction, and peripheral artery disease. Atherosclerosis is caused by an accumulation of foam cells, which causes partial or complete arterial stenosis. It has long been known that HDL protect against the formation of atherosclerotic lesions. This has been attributed in part to the antioxidant and anti-inflammatory properties of HDL. However, the main protective effect of HDL is due to their maintenance of cellular cholesterol homeostasis via RCT, a process by which HDL recover excess cholesterol in cells and transport it back to the liver to be eliminated in the feces.

RCT is a multi-step process that is initiated by the efflux of cellular cholesterol to lipid-poor apoA-1 via an interaction with the ABCA1 transporter. There is increasing evidence indicating that other HDL-associated proteins, including PON1, may interact with the ABCA1 transporter on macrophages to initiate cholesterol efflux to HDL [8]. Different mechanisms have been proposed to explain the mechanisms by which PON1 may stimulate or mediate cholesterol efflux from macrophages [8,16]. However, all the studies to date have been conducted in vitro using recombinant or plasma PON1 [8,16]. The present study investigated the effect of overexpressing human PON1 in mice on the RCT process. Our results showed that the overexpression of PON1 in mice substantially promotes the excretion of macrophage-derived cholesterol in the feces. In addition, our ex vivo experiments showed that MPM from PON1-Tg mice liberate more cholesterol than MPM from WT mice and that HDL-mediated cholesterol efflux is higher in PON1-Tg mice than in WT mice. Transfection of J774 macrophages with human PON1 also stimulates their capacity to release cholesterol excess. These results provided new evidence that the overexpression of PON1 promotes cholesterol homeostasis by increasing RCT and macrophage cholesterol efflux.

Cholesterol efflux is the first and rate-limiting step of RCT in which intracellular cholesterol from macrophages is transferred to apoA-1 and HDL mainly via the ABCA1 and ABCG1 transporters. Cholesterol efflux depends on the capacity of macrophages to liberate cholesterol and apoA-1 and HDL to accept cholesterol. Our results showed that the overexpression of PON1 in mice enhances [^3^H]-cholesterol efflux from MPM to HDL. In terms of the mechanisms by which PON1 may stimulate cholesterol efflux in vitro, Berrougui et al. showed that PON1 upregulates *ABCA1* mRNA and protein expression by J774 macrophages [8]. The effect of PON1 has been attributed to its phospholipase A2-like activity, which contributes to the formation of LysoPC which, in turn, stimulates ABCA1 expression [8,25]. This is supported by the results of the present study, which showed that MPM from PON1-Tg mice express higher levels of ABCA1 than MPM from WT mice.

ABCA1 is ubiquitously expressed in human and murine tissues [26,27] and plays an important role in HDL biogenesis. ABCA1 mediates the cellular efflux of phospholipids and cholesterol to lipid-free apoA-1, contributing to the generation of pre-β-HDL and discoidal HDL particles, the first steps of HDL metabolism. The expression of ABCA1 is regulated by the PPARγ/LXRα pathway. The transcriptional regulation of ABCA1 is mediated by LXRα, which is regulated by PPARγ [28,29,30]. The PPARγ/LXRα/ABCA1 pathway is crucial for the regulation of the efflux of cholesterol from peripheral cells [31]. Wang et al. and Joseph et al. showed that the PPARγ/LXRα/ABCA1 pathway is activated by natural or synthetic agonists, which upregulates ABCA1 expression, enhances cell cholesterol efflux, and reduces atherosclerosis development [32,33]. Conversely, the inhibition of PPARγ and LXRα results in a decrease in the expression of the ABCA1 transporter [34]. Interestingly, our results showed that MPM from PON1-Tg mice express higher levels of ABCA1, PPAR*γ*, and LXR*α* mRNA and protein than MPM from WT mice. These same results were obtained with J774 macrophages transfected with human PON1, which provides a support for the assumption that PON1 is involved in the in vivo stimulation of the expression of ABCA1 on macrophages via the regulation of the PPARγ/LXRα/ABCA1 pathway.

The ability of HDL particles to transport cholesterol from peripheral cells such as lipid-loaded macrophages also affects the RCT process. Our results showed that HDL from PON1-Tg mice can accept more cholesterol from J774 macrophages than HDL from WT mice and that this capacity is significantly enhanced in the presence of ABCA1-enriched J774 macrophages. These results are in agreement with previous in vitro studies showing that purified human plasma and recombinant PON1 both stimulate the cholesterol efflux capacity of HDL and that this effect is significantly related to the expression levels of the ABCA1 transporter [8]. ApoA-1 has been identified as the major HDL-associated protein that interacts with the ABCA1 and ABCG1 transporters and the SR-BI receptor, enabling the initiation of cellular cholesterol efflux. However, other HDL-associated proteins may also posses this function, especially proteins that contain multiple amphipathic helical domains [35]. It has been suggested that PON1 may be an HDL-associated protein that possesses this function [8]. The secondary structure of PON1 contains amphipathic helices with approximately 22 amino acids that are required for the interaction with the ABCA1 transporter [36]. PON1 reacts via an apoA-1-like mechanism and mediates cholesterol efflux from macrophages through a fast cholesterol efflux phase via the ABCA1 transporter and a slower cholesterol efflux phase involving the ABCG1 transporter and the SR-BI receptor [8,37]. Unlike apoA-1, which allows the efflux of cholesterol only to lipid-free and lipid-poor apoA-1, PON1 may thus stimulate the efflux of cholesterol to nascent (pre-β-HDL) and mature HDL (HDL2 and HDL3) particles [8,37].

Our results showed that overexpression of PON1 in mice do not affect the plasma HDL levels, which is in agreement, with those obtained by Tward et al. [14]. As such, the increase in the cholesterol efflux capacity of HDL from PON1-Tg mice may be due to an improvement in HDL functionality rather than HDL concentration. The increase in the plasma paraoxonase activity of PON1-Tg mice may reflect an increase in the PON1 protein content of their HDL, which would explain the improvement in their capacity to mediate cholesterol efflux as previously suggested [8]. Our results confirmed those of Zhu et al., who suggested that the modulation of PON1 activity may be a potential novel mechanism for the regulation of HDL-mediated cholesterol efflux capacity [38].

The in vivo measurement of the effect of PON1 on cholesterol homeostasis showed that the overexpression of PON1 in mice significantly increased the RCT process as shown by the level of [^3^H]-cholesterol-derived macrophages in the feces. The levels of [^3^H]-cholesterol-derived macrophages at each step in the RCT process, that is, in the plasma, liver, and feces, clearly confirmed that there is an increase in cholesterol transfer from [^3^H]-cholesterol-loaded macrophages to the plasma and liver and then to the feces. The increase in [^3^H]-cholesterol levels in the plasma confirmed the in vitro measurements showing that HDL from PON1-Tg mice have an increased capacity to mediate cholesterol efflux compared to HDL from WT mice. It is worth noting that the improvement in cholesterol efflux through the RCT pathway is thought to mainly protect against CVD by removing cholesterol from foam cells in atherosclerotic plaque [3,6,39]. Previous studies aimed at elucidating the role of PON1 in the atherosclerotic process have shown that PON1-deficient mice (PON1^-^/^-^) are more susceptible to developing atherosclerosis [13] and that mice that overexpress PON1 are less susceptible to atherosclerosis than WT mice [14]. Interestingly, both studies focused mainly on the anti-inflammatory and antioxidant properties of PON1 to explain the difference in the susceptibility to atherosclerosis of PON1^-^/^-^ and PON1-Tg mice and paid little attention to the role of PON1 in the regulation of RCT. Our study is the first to show that the overexpression of PON1 in mice significantly increases the transfer of cholesterol to the feces via the stimulation of RCT and to propose a novel mechanism for the anti-atherosclerotic effect of PON1.

The effect of overexpressing proteins involved in HDL biogenesis, especially apoA-1 and ABCA1, on the development of atherosclerosis has been the subject of several investigations [40–42]. The overexpression of apoA-1 in mice has been associated with a significant reduction in the atherosclerotic process [23,41,43,44]. In addition, Singaraja et al. showed that the overexpression of ABCA1 significantly reduces the formation of atherosclerotic lesions in apoE-deficient mice [42]. Interestingly, this protective effect has been attributed to the stimulation of the HDL-mediated cholesterol efflux capacity and an improvement in the RCT process resulting from the overexpression of apoA-1 and ABCA1 [41,42]. These results are consistent with those of Tward et al., who showed that mice overexpressing PON1 are less susceptible to atherosclerosis [14]. Our results suggest that this beneficial effect may be mainly due to the increased capacity to up-regulate HDL-mediated cholesterol efflux and the RCT process.

In summary, our results showed that the overexpression of PON1 in mice significantly increases cholesterol efflux from macrophages due to an increase in the cholesterol efflux capacity of HDL and the stimulation of ABCA1 expression on macrophages. Our results also showed, for the first time, that PON1 overexpression promotes in vivo macrophage-specific RCT and enhances cholesterol elimination in the feces. Given the importance of the stimulation of RCT with respect to cardiovascular protection, our results provide new evidence to help explain the mechanism of the anti-atherogenic effect of PON1.
